# Attitudes Toward Lesbians and Gay Men Scale: validation in Brazilian physicians

**DOI:** 10.31744/einstein_journal/2019AO4527

**Published:** 2019-04-25

**Authors:** Renata Corrêa-Ribeiro, Fabio Iglesias, Einstein Francisco Camargos

**Affiliations:** 1 Universidade de Brasília, Brasília, DF, Brazil.

**Keywords:** Homosexuality, Prejudice, Factor analysis, statistical, Psychometrics, Physicians, Translating, Brazil, Homossexualidade, Preconceito, Análise fatorial, Psicometria, Médicos, Tradução, Brasil

## Abstract

**Objective:**

To perform the cross-cultural adaptation of the original North American version of the Attitudes Toward Lesbians and Gay Men Scale (ATLG) for use in Brazil, and to evaluate the psychometric properties of the adapted instrument in a sample of Brazilian heterosexual physicians.

**Methods:**

Stages of cross-cultural adaptation were as follows: translation by two independent evaluators, translation synthesis, evaluation by the target population for semantic equivalence, pilot study with 42 physicians, and final instrument preparation involving 224 heterosexual physicians practicing medicine in the Federal District. Invitations containing a link to the study were sent to physicians via e-mail, social media and medical associations. Inclusion criteria were as follows: being a physician authorized to practice medicine in Brazil, practicing medicine in the Brazilian Federal District, and self-declared heterosexual, as stipulated in the original version of the ATLG scale. Exclusion criteria were not disclosed to potential participants to avoid inhibition and unwillingness to participate; respondents meeting exclusion criteria were removed from the sample during data analysis.

**Results:**

Exploratory and confirmatory factor analyses suggested a one-factor solution to be the most representative of the data, including all 20 items with high reliability (composite reliability coefficients =0.948).

**Conclusion:**

The ATLG scale is a suitable instrument to assess physicians’ attitudes toward homosexuals, with good validity and reliability evidence based on the sample studied.

## RESUMO

**Objetivo:**

Realizar a adaptação transcultural da versão original norte-americana do inventário *Attitudes Toward Lesbian and Gay Men Scale* (ATLG) sobre atitudes diante de lésbicas e homens gays para uso no Brasil, e avaliar as propriedades psicométricas em uma amostra de médicos heterossexuais brasileiros.

**Métodos:**

Foram realizadas as seguintes etapas de adaptação transcultural: tradução por dois avaliadores independentes, síntese das traduções, avaliação pela população-alvo para equivalência semântica, estudo piloto com 42 médicos, e processo final de elaboração do instrumento, envolvendo 224 médicos heterossexuais que exerciam medicina no Distrito Federal. Um convite contendo um *link* para o estudo foi enviado aos médicos por *e-mail*, por meio de redes sociais e de associações médicas. Os critérios de inclusão foram: ser médico atuante, exercer a atividade no Distrito Federal e se autodeclarar heterossexual (conforme estipulado na ATLG). Para evitar a inibição ou impedir a resposta, os critérios de exclusão não foram divulgados aos potenciais participantes. Questionários que preencheram critérios de exclusão foram removidos da amostra durante a análise de dados.

**Resultados:**

Análises fatoriais exploratórias e confirmatórias sugeriram um único fator como sendo o mais representativo para os dados, reunindo os 20 itens com fidedignidade elevada (coeficiente de confiabilidade composta = 0,948).

**Conclusão:**

A ATLG é um instrumento adequado para avaliar as atitudes dos médicos em relação aos homossexuais, com evidência de validade e confiabilidade na amostra analisada.

## INTRODUCTION

In the United States, about 2.4% of interviewees declared themselves as lesbian, gay or bisexual.^(^ ^1^ ^)^ The Brazilian census does not ask about sexual orientation and only data on same-sex couples are available. According to the *Instituto Brasileiro de Geografia e Estatística* (IBGE), the number of homosexual couples amounted to 60 thousand in 2010.^(^ ^2^ ^)^

In 1973, homosexuality ceased to be considered a mental disorder by the American Psychiatry Association (APA); still, discrimination against gay men and lesbians is recognized as a potential barrier in physician-patient relationships.^(^ ^3^ ^,^ ^4^ ^)^ Also, compared to heterosexuality, homosexuality is associated with differences in morbidity and risk factors for several diseases, such as higher smoking rates,^(^ ^5^ ^-^ ^7^ ^)^ excessive alcohol use and greater tendency toward obesity,^(^ ^5^ ^-^ ^8^ ^)^ increased frequency of drug use,^(^ ^5^ ^)^ higher prevalence of cardiovascular diseases,^(^ ^7^ ^)^ and greater likelihood of developing breast cancer.^(^ ^8^ ^,^ ^9^ ^)^

According to the position statement developed by the American Geriatrics Society (AGS) committee in 2015, discrimination faced by Lesbian, Gay, Bisexual or Transgender (LGBT) individuals when seeking health care is evidenced by the denial of certain medical care services, generalized heteronormative assumptions, refusal to accept a chosen caregiver during hospitalization and overt derogatory statements that may lead to health care seeking delay or avoidance out of fear of discrimination.^(^ ^10^ ^,^ ^11^ ^)^

Brazilian studies investigating physicians’ attitudes toward or overt prejudice against homosexual patients, or how this construct interferes with the quality of medical care provided, are lacking. This gap is in part due to the lack of specific validated instruments aimed to assess physicians’ attitudes toward homosexuals. Moreover, these instruments must address attitudes toward homosexuals only, excluding other sexual and gender minorities, such as transgender, if they are to distinguish attitudes toward lesbians and gay men. Specificity is required because physicians may deal with only one of these groups in clinical practice (*e.g*., lesbians and gynecologists) and due evidences pointing to considerable differences in attitudes toward lesbians and gay men.^(^ ^12^ ^)^

## OBJECTIVE

To perform the cross-cultural adaptation of the original North American version of the Attitudes Toward Lesbians and Gay Men Scale for use in Brazil, and to evaluate the psychometric properties of the adapted instrument, based on a sample of heterosexual Brazilian physicians.

## METHODS

### Participants

Participants were recruited using snowball sampling. Research advertising and invitations for voluntary participation were sent to physicians via e-mail, social media and professional organizations. The following inclusion criteria were applied to the initial sample: being a physician authorized to practice Medicine in Brazil; practicing medicine in the Brazilian Federal District; and heterosexual, as stipulated in the original version of the scale. Sample size was calculated based on the suggested ratio of ten participants per observable variable. Hence, given the scale consisted of 20 items, a minimum sample size of 200 valid cases was required. After completion of the study, the need to evaluate 100 participants per factor was also met.^(^ ^13^ ^)^ The final sample comprised 224 heterosexual physicians acting medicine in Brazil, including 149 women (66.5%).

### Instrument

The Attitudes Toward Lesbians and Gay Men Scale (ATLG) is an instrument designed to measure the attitudes of heterosexuals toward lesbians and gay men.^(^ ^12^ ^)^ A major advantage of ATLG is that it can be divided into two subscales for separate assessment of attitudes toward lesbians and gay men. Given the inclusion of gynecologists (*i.e*., health professionals dealing exclusively with women) in the sample, such differentiation was essential. The fact that ATLG was considered one of the best instruments from a validity and reliability standpoint, in a systematic review of instruments designed to assess homophobia and related constructs,^(^ ^14^ ^)^ was yet another reason for choosing this particular instrument.

The original ATLG consists of 20 items, the first 10 about lesbians and the next 10 about gay men, and can be used as a single scale or two distinct subscales (Attitudes Toward Lesbians and Attitudes Toward Gay Men). Items are scored using a 9-point Likert scale, end points being “strongly disagree” and “strongly agree”. The total scale score ranges from 20 to 180, with higher scores indicating more negative attitudes. Subscale scores range from 10 to 90. The ATLG was validated in several countries, including China,^(^ ^15^ ^)^ the Netherlands,^(^ ^16^ ^)^ and Argentina.^(^ ^17^ ^)^

### Procedures

#### Stages of cross-cultural adaptation

This study followed all procedures required to ensure content of the original instrument: translation from the source language (English) to the target language (Portuguese) by two evaluators; synthesis of the translated versions; analysis of the final version by experts, semantic evaluation by the target population; and finally, a pilot study.^(^ ^18^ ^)^

Two independent translations of the scale were produced by two native speakers of Portuguese (target language) who were fluent in English (source language): an experienced researcher in cross-cultural adaptation of instruments, and a person not involved in academic work. A synthesis of these translations was made by a group of professors with solid knowledge of cross-cultural adaptation of instruments. This was followed by a discussion of the final version of the adapted instrument for implementation by the authors and a psychometrics professional.

The final version of the adapted instrument was evaluated by 13 physicians who completed the questionnaire and were asked to examine the items for clarity. Suggestions made by the target population were then evaluated, leading to changes in items 4, 8 and 17 of the adapted scale. Item 4 was thought to be the least clear; the original statement was translated as “*Leis estaduais que regulam o comportamento lésbico consensual e privado deveriam ser flexibilizadas*” (“State laws regulating private, consenting lesbian behavior should be loosened”). Although semantic equivalence between the original and translated versions was maintained, unlike the United States, there are no state laws regulating homosexual behavior in Brazil. Therefore, the word “state” was removed from the sentence, resulting in “Laws regulating private, consenting lesbian behavior should be loosened”. Still, doubts as to the adequacy of the item to the Brazilian context remained. In item 8, elimination of the word “basic” was suggested, resulting in “Female homosexuality is a threat to many of our social institutions”. Finally, the elimination of the word “too” in item 17 was also suggested, resulting in “I would not be upset if I learned my son is a homosexual”.

#### Steps of the quantitative process

After implementation of above described modifications, the final version of the instrument was uploaded into an online survey platform for a quantitative pilot study with 42 heterosexual physicians. In addition to the adapted final version of the ATLG, a questionnaire aimed to assess participants’ knowledge about homosexuality (results of this questionnaire will be submitted in a separate manuscript) and collecting demographic data were also made available on the online platform. The following demographic data were collected: age (years), sex (male/female), religion (Catholic, Evangelical, Spiritist, none or other), marital status (single, married/consensual union, separated/divorced or widowed), professional status (practicing physician, resident physician, retired or not practicing) and sexual orientation (heterosexual, homosexual, bisexual, none of the above, I do not know or other).

Data were collected from March to September 2016. The study was approved by the Research Ethics Committee of the *Universidade de Brasília*, under official opinion number 1.339.076, CAAE: 49275115.0.0000.5558. Requirements for informed consent were waived since it was felt the process of obtaining consent might inhibit participation, or allow the identification of participants. Use of a limited set of sociodemographic data helped protect anonymity of research participants, even from the authors.

### Statistical analysis

Descriptive statistics and exploratory factor analysis were used to identify the factor structure of the instrument. The number of factors to be retained in the instrument was determined using the Hull method^(^ ^19^ ^)^ and Factor version 10 software.^(^ ^20^ ^)^ Instrument reliability was investigated using composite reliability coefficients, with values greater than 0.70 indicating satisfactorily relation between scale items. Items with factor load <0.30 were defined as exclusion criteria.^(^ ^21^ ^)^

Confirmatory factor analysis was performed using Mplus software. Weighted least squares-mean and variance-adjusted (WLSMV) estimation was performed using a polychoric correlation matrix, and respecting the ordinal nature of the data. The following fit indices were evaluated: root mean square error of approximation (RMSEA), comparative fit index (CFI) and Tucker-Lewis index (TLI). Root mean square error of approximation values <0.08 were expected within a confidence interval (90%) not greater than 1.00, along with CFI and TLI values >0.90 (preferably >0.95).

## RESULTS

The final sample consisted of 224 heterosexual physicians aged 24 to 72 years (mean age, 42.2 years; standard deviation − SD of 9.5) and practicing medicine in the Federal District, including 149 (66.5%) women. Of these, 208 (92.9%) were practicing physicians and 16 (7.1%) resident physicians. Physicians in this sample were either Catholic (102; 45.5%), Spiritist (41; 18.3%), Evangelical (27; 12.1%), nonreligious (44; 19.6%) or had other religions (10; 4.4%). Marital status was stratified as married or living in consensual union (174; 77.7%), single (31; 13.8%), separated or divorced (18; 8.0%) or widowed (1; 0.4%).

The sample was thought to be amenable to exploratory factor analysis (Kaiser-Meyer-Olkin − KMO − coefficient of 0.956). Percentage of explained variance, scree plot and eigenvalue analysis suggested the extraction of up to three factors from the ATLG scale. The Hull method suggested a one-factor solution as the most representative of the data, including all 20 items with high reliability (composite reliability coefficients, c=0.948). Items in the female and male homosexuality scales did not diverge; therefore, perceptions of female and male homosexuality did not differ significantly. Factor loadings of scale items are shown in table 1.

**Table 1:** Structure and factor loadings of the translated and adapted version of the Attitudes Toward Lesbian and Gay Men Scale

Items	Factor loading
1. Lesbians just can't fit into our society	0.673
2. A woman's homosexuality should not be a cause for job discrimination in any situation*	-0.311
3. Female homosexuality is detrimental to society because it breaks down the natural divisions between sexes	0.815
4. Laws regulating private, consenting lesbian behavior should be loosened*	-0.429
5. Female homosexuality is a sin	0.762
6. The growing number of lesbians indicates a decline in the moral values of society	0.806
7. Female homosexuality in itself is no problem unless society makes it a problem*	-0.376
8. Female homosexuality is a threat to many of our social institutions	0.753
9. Female homosexuality is an inferior form of sexuality	0.759
10. Lesbians are sick	0.666
11. Male homosexual couples should be allowed to adopt children the same as heterosexual couples*	-0.639
12. I think male homosexuals are disgusting	0.732
13. Male homosexuals should not be allowed to teach school	0.638
14. Male homosexuality is a perversion	0.865
15. Just as in other species, male homosexuality is a natural expression of sexuality in human men*	-0.746
16. If a man has homosexual feelings, he should do everything he can to overcome them	0.816
17. I would not be upset if I learned that my son is a homosexual*	-0.545
18. Homosexual behavior between two men is just plain wrong	0.885
19. The idea of male homosexual marriages seems ridiculous to me	0.856
20. Male homosexuality is merely a different kind of lifestyle that should not be condemned*	-0.527
KMO	0.956
Bartlett's sphericity test	2,890.81†
Explained variance	51.01%
Composite reliability coefficients	0.948

Version tranlated and adapted from Herek GM. Heterosexuals’ attitudes toward lesbian and gay men: correlates and gender differences. J Sex Res. 1988;25(4):451-77.^(^ ^12^ ^)^

* Reversed items;

† p<0.01.

Confirmatory factor analysis revealed acceptable fit indices for the exploratory model (Table 2). Fit indices were also investigated for a model considering male and female dimensions separately. The latter fit indices were also thought to be acceptable; however, the 0.937 correlation between factors suggested both dimensions were in fact part of a single construct and the one-factor solution should be maintained.

**Table 2:** Confirmatory factor analysis of competing models of the adapted version of the Attitudes Toward Lesbian and Gay Men Scale

Model	χ^2^ *	df	χ^2^/df†	RMSEA	CFI	TLI
Exploratory	271.645	170	1.60	0.052 (0.040-0.063)	0.943	0.936
Female homosexuality assessment	39.364	35	1.12	0.024 (0.000-0.055)	0.992	0.990
Male homosexuality assessment	79.799	35	2.28	0.076 (0.054-0.098)	0.942	0.926

* likelihood-ratio χ^2^;

† normalized χ^2^.

df: degrees of freedom; RMSEA: root mean square error of approximation; CFI: comparative fit index; TLI: Tucker-Lewis index.

Finally, specific analyses were performed for the male and female homosexuality dimensions to investigate whether these dimensions would be effective as a single instrument aimed at assessing attitudes toward a specific gender (*i.e*., using subscales independently). Results revealed acceptable fit indices for both subscales; however, the female dimension was better fitted than the male dimension.

## DISCUSSION

Given the lack of a specific questionnaire for separate assessment of attitudes of heterosexual physicians toward gay men and lesbians in Brazil, this study set out to adapt the ATLG scale and gather validity and reliability evidences. Homosexuality ceased to be considered a disease since 1973; still, it is a critical vulnerability factor requiring proper recognition and effective, specific management on the part of health professionals. The fact that sexual minorities may avoid health care seeking out of fear of disrespectful behavior and discriminatory treatment may partially explain the increased prevalence of diseases and risk factors in these patients.^(^ ^22^ ^,^ ^23^ ^)^

In spite of the growing numbers of Brazilian social science studies addressing prejudice, homophobia and attitudes toward homosexuals over the last 10 years, these are mostly focused on undergraduate students^(^ ^24^ ^,^ ^25^ ^)^ and therefore of limited representativeness. In addition, the instruments used in some of these studies were not cross-culturally adapted or validated for use in Brazil.

According to Costa et al.,^(^ ^26^ ^)^ the distinction between manifestations of prejudice against sexual orientation and gender expression seems to be very subtle in Brazil. For this reason, an instrument that accounts for this cultural specificity (*i.e*., that does not distinguish non-heterosexual orientation from gender expressions that do not fit the conventional norms of masculinity and femininity) was developed.^(^ ^26^ ^)^ Hence, despite the clear distinction between homosexual orientation (which reflects desire) and gender expressions that are perceived as different from the norm (transgender/transsexual identity), this distinction may not be obvious to those manifesting prejudice.

Contrary to this belief, which derives from samples of the general population and undergraduate students in particular, in health care settings the physician-homosexual patient relationship tends to be based on self-reported sexual orientation rather than phenotypic characteristics or “boyish/girlish behavior”. Homosexual patients who fit the characteristics associated with their sex role (masculine men and feminine women), who may benefit from a certain degree of social invisibility and therefore be spared from discriminatory attitudes, often experience discrimination in medical consultations. Once the sexual orientation of the patient is revealed, prejudice and lack of knowledge become apparent in statements such as, “How come a beautiful woman such as yourself, who could have any man you wanted, chooses to be a lesbian?”. Given the power nature of physician-patient relationships, the assessment of constructs, such as sex-related attitudes and prejudice, must account for related peculiarities. The cross-cultural adaptation of the ATLG scale for use in Brazil may help address the specificities of this particular physician-patient relationship.

Good evidence for validity of the ATLG can be found in the current literature. Moreover, the ATLG is the most commonly used tool in research with physicians and health professionals in the English language. The first study evaluating the psychometric properties of this instrument was conducted by Herek, in 1988, and gathered evidence of validity in three distinct samples of students, with alpha values of 0.90, 0.95 and 0.92.^(^ ^12^ ^)^ Similar alpha values (0.94) were found in this study.

In a study describing the use and adaptation of the ATLG to the Dutch population, factorial analysis also revealed a single factor encompassing all questionnaire items. According to authors of that study, some items in the North American version required cultural adaptation, given the higher tolerance of homosexuality among Dutch compared to American people. Item modifications were based on rating of terms such as “sick” and “disgust” as “strong” or “extreme” for the Dutch context. This shows that, although semantic properties allow instrument adaptation to different primary cultures, some modifications are required for cultural context assessment. As in this study, the item referring to “state laws” also had to be modified.^(^ ^27^ ^)^

This study had similar limitations to other research investigating prejudice, including a relatively low rate of response to invitations to participate, and potential impacts of social desirability phenomena, such as providing socially acceptable responses that are probably not true. Given the snowball sampling method used in the study, response rates could not be estimated. Finally, future research is warranted to test for convergent and predictive validity. We hope this study will help to fill a major gap in the Brazilian literature regarding the availability of instruments aimed to measure prejudice against homosexuals, and serve to assess physicians practicing in other locations, as well as other healthcare professionals and professionals from other areas, such as education, thereby promoting improvements in care delivered to sexual minorities.

## CONCLUSION

Based on results of this study, the Attitudes Toward Lesbians and Gay Men Scale is a suitable instrument to assess physicians’ attitudes toward homosexuals, with evidence for validity in the sample studied.
